# Challenges of medical student underperformance

**DOI:** 10.3402/meo.v19.26041

**Published:** 2014-11-24

**Authors:** Rizwan Dewji, Dushyanth Gnanappiragasam, Abbas Dewji

**Affiliations:** 1 Imperial College School of Medicine, Imperial College London, London, SW7 2AZ, UK; 2 Imperial College School of Medicine, Imperial College London, London, UK

We read the article by Stratton and Elam (1) with interest and believe that it raises some important points in relation to the performance of medical students. The article concluded that a number of factors, including a lower undergraduate science grade point average entering medical school via an accelerated BS/MD track and being over the age of 31, were associated with first-year academic underperformance.

We believe that the research conducted is not only insightful but also sheds light on what is a relatively under-researched area. There are a number of reasons why we believe that research into the appropriate selection and subsequent performance of first-year medical students is of optimum importance. Jones and Korn (2) found the cost of medical student education to be US$40,000–50,000 per student, per year in 1997, with the costs likely to have increased in the subsequent 17 years. Furthermore, with medical student attrition rate estimated at 14% (3), a significant amount of resources are being wasted by poor medical student selection, and anything that can be done to reduce this attrition rate, and hence the inappropriate use of resources should be encouraged. In addition to the financial cost, there is a social and personal cost to medical student underperformance with individuals being subject to unnecessary stress as well as having to divert career paths.

Whilst the results are undoubtedly useful, we propose that they should be used with caution. In particular, selection panels should avoid discrimination against those who are at ‘higher risk’ of underperformance. For example, although the study concludes that students over the age of 31 are more likely to underperform in their first year of medicine, our personal experience has suggested that these students often have a greater level of commitment and motivation to study and succeed in the medical profession.

Hence, in order to further validate and investigate this important area, we believe that additional research should be conducted. In particular, it would be beneficial to assess medical student underperformance in a wider range of medical schools to establish whether the suggested factors for underperformance are applicable in a wider context. Additionally, research into variables which are associated with improved performance in medical school would also be of interest and may aid the medical student selection process.

*Rizwan Dewji* Imperial College School of Medicine Imperial College London London SW7 2AZ UK Email: rizwan.dewji09@imperial.ac.uk *Dushyanth Gnanappiragasam* Imperial College School of Medicine Imperial College London London UK *Abbas Dewji* Imperial College School of Medicine Imperial College London London UK
